# Analysis of the PI3K-AKT-mTOR pathway in penile cancer: evaluation of a therapeutically targetable pathway

**DOI:** 10.18632/oncotarget.24688

**Published:** 2018-03-23

**Authors:** Anthony Adimonye, Elzbieta Stankiewicz, Sakunthala Kudahetti, Giorgia Trevisan, Brendan Tinwell, Cathy Corbishley, Yong-Jie Lu, Nick Watkin, Daniel Berney

**Affiliations:** ^1^ Centre for Molecular Oncology, Bart’s Cancer Institute, Barts and The London School of Medicine and Dentistry, Queen Mary University of London, London, United Kingdom; ^2^ Department of Histopathology, Royal London Hospital, Barts Health NHS Trust, London, United Kingdom; ^3^ Department of Cellular Pathology, St George’s Hospital, London, United Kingdom; ^4^ Department of Urology, St George’s Hospital, London, United Kingdom

**Keywords:** phosphatidylinositol-4,5-bisphosphate 3- kinase, catalytic subunit alpha, penile squamous cell carcinoma, copy number gain, p-AKT

## Abstract

**Objectives:**

To determine whether phosphatidylinositol-4,5-bisphosphate 3- kinase, catalytic subunit alpha (PIK3CA) copy number gain is common and could prove a useful marker for the activation status of the PI3K-AKT-mTOR pathway in penile squamous cell carcinoma (PSCC).

**Methods:**

Fresh frozen tissue and archival blocks were collected from 24 PSCC patients with 15 matched normal penile epithelium (NPE) tissue from St George’s Hospital. PIK3CA mutational and copy number status (CNS) was assessed via Sanger sequencing and fluorescence *in-situ* hybridisation, respectively. PIK3CA RNA expression was quantified using TaqMan gene expression assay. HPV DNA was detected with INNO-LiPA assay. p-AKT and p-mTOR protein expression were assessed using western blot and immunohistochemistry.

**Results:**

PIK3CA copy number gain was found in 11/23 (48%) patients, with mutations present in only 2/24 (8%) patients. In comparison to NPE, PSCC showed significantly lower PIK3CA RNA expression (p=0.0007), p-AKT (Ser473) nuclear immunoexpression (p=0.026) and protein expression of p-AKT (Thr308) (p=0.0247) and p-mTOR (Ser2448) (p=0.0041). No association was found between PIK3CA CNS and p-AKT and p-mTOR protein expression.

**Conclusion:**

Based on our results the PI3K-AKT-mTOR pathway is not a key driver in PSCC carcinogenesis and the therapeutic targeting of this pathway is unlikely to produce significant clinical benefit.

## INTRODUCTION

Penile cancer is a rare malignancy in the developed world representing 0.3 −0.5% of all male malignancies in Europe and USA and 95% of cases are classified as squamous cell carcinoma of the penis (PSCC) [1, 2]. Several risk factors have been identified which include high-risk human papilloma virus (Hr-HPV), phimosis, trauma and smoking among others [2, 3].

Advanced PSCC, which can be highly aggressive with a significant propensity to metastasise [4, 5], remains difficult to manage as most are chemo/radio-resistant with limited treatment options available, when first line chemotherapeutic options fail [6, 7]. Few targeted therapeutic agents have been used as second-line treatment for patients with refractory disease with modest results such as anti-epidermal growth factor receptor monoclonal antibodies such as cetuximab [8–10], and receptor tyrosine kinase inhibitors such as sorafinib [11]. There is a clinical need for new therapeutics targets and agents.

**Figure 1:**
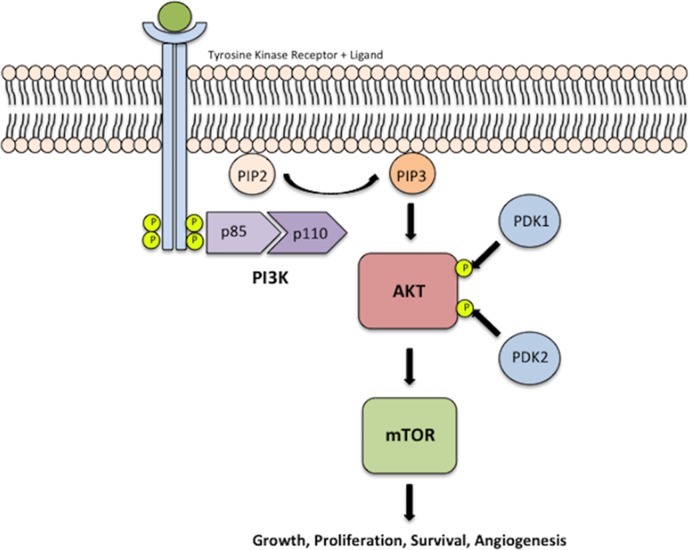
PI3K-AKT-mTOR pathway Activation of PI3K leads to phosphorylation of the p85 subunit of the PI3K heterodimer, which subsequently activates the catalytic subunit, p110. This phosphorylated heterodimer catalyses the conversion of PIP_2_ into PIP_3._ PIP_3_ mediates the activation of AKT via phosphorylation by PDK-1 at threonine T308 and PDK-2 (mTOR2) at serine residue S473, for maximal AKT activation. Activated AKT then mediates the activation of mTOR, which results in cellular growth, survival, angiogenesis and proliferation.

The deregulated PI3K-AKT-mTOR pathway plays a pivotal role in many human cancers leading to cellular proliferation, survival and angiogenesis [12, 13] (Figure 1). Licensed treatment options targeting this pathway currently exist such as mTOR inhibitors, everolimus and temsirolimus, which have both been used in renal cell carcinoma among other tumours [14]. In addition a vast number of other biological therapies targeting this pathway are currently in clinical trials in multiple advanced solid tumours including epithelioid squamous cell tumours such as cervical [15, 16] and head and neck cancer [17, 18]. This opens the potential for the use of current and/or newer treatments targeting this pathway in PSCC.

Multiple studies have taken place looking into the role of the PI3K-AKT-mTOR pathway in penile carcinogenesis. It was suggested that EGFR, HER3 and HER4 overexpression is associated with penile carcinogenesis [3]. Moreover, the phosphatidylinositol-4,5-bisphosphate 3- kinase, catalytic subunit alpha (PIK3CA) oncogene was found to be mutated in 8/28 (29%) penile cancer cases by Andersson *et al*. [19] and Ferrandiz-Pulido *et al*. [20] more recently found a lower prevalence of PIK3CA mutations in 6/65 (9%) PSCC cases. Ferrandiz-Pulido *et al*. [21] in 67 PSCC cases found phospho-mTOR (activated mTOR) and phospho-elF4E (a downstream effector protein of mTOR) immunoexpression was significantly increased in PSCC compared to adjacent normal tissues and associated with lymph node metastasis (p=0.05 and p=0.006, respectively).

In addition, recent work by our group, La-Touche *et al*. [22] found copy number gains in 55/64 (86%) cases within the 3p12.3-q29 loci, similar to Busso-Lopes *et al*. [23] in penile cancer via array comparative genomic hybridisation (aCGH). Within this chromosomal region resides the PIK3CA oncogene, at the 3q26.3 locus, which is possibly the driver gene for this copy number aberration.

Our main objective was to determine whether PIK3CA copy number gain is common in PSCC and whether this aberration could prove a useful marker for the activation of the PI3K-AKT-mTOR pathway and thus the potential use of biological therapies to target this pathway in advanced PSCC.

## RESULTS

We analysed 24 patients with primary PSCC with a median age of 66 (range 35 - 86), of which 15 had matched normal penile epithelium (NPE). Their histopathological characteristics, Hr-HPV status, PIK3CA CNS and Mutational status are detailed in Table 1.

**Table 1:** Patient and tumour characteristics of PSCC cohort

Patient and Tumour Characteristics	
	**N (%)**
**Age**	
Median (Range)	66 (35 - 86)
**Tumour Grade**	
Intermediate	5 (21%)
High	19 (79%)
**Tumour Stage**	
Ia Ib	2 (8%) 1 (4%)
IIa IIb	7 (29%) 2 (8%)
IIIa IIIb	8 (34%) 1 (4%)
IV	3 (13%)
**Nodal Status^#^**	
Negative	9 (45%)
Positive	11 (55%)
**Hr-HPV Status**	
Negative	7 (29%)
Positive	17 (71%)
PIK3CA CNS^*^	
No Gain	12 (52%)
Gain	11 (48%)
**PIK3CA Mutational Status**	
WT	22 (92%)
Mutated	2 (8%)

^#^ - Nodal status not available for 4 PSCC cases; ^*^ - Poor FISH signal for one PSCC case so excluded.

### PIK3CA CNS and mutational analysis

**Figure 2:**
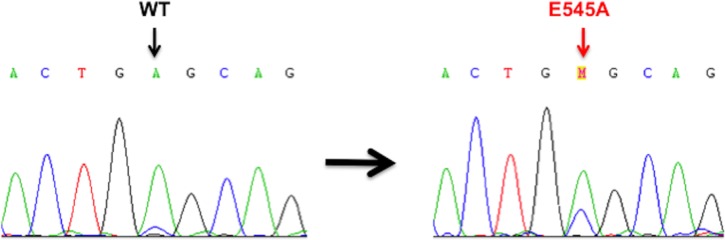
Sanger sequencing electropherograms of Wild-Type (WT) and mutant PIK3CA in the primary PSCC cases E545A mis-sense mutation in exon 9 of PIK3CA WT (GAG) → Mutant (GCG) found in two PSCC cases.

**Figure 3:**
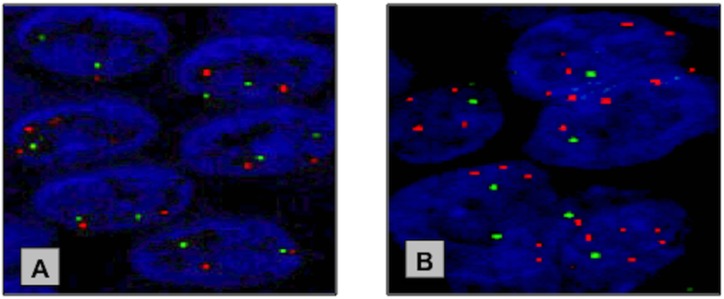
Representative FISH images of the copy number changes at the PIK3CA locus in NPE and PSCC specimen **(A)** Neutral copy number at PIK3CA locus in NPE. **(B)** PIK3CA copy gain in primary PSCC. Red Probe – *PIK3CA* locus (3q26.3) & Green Probe - control locus (3p21.3).

PIK3CA mutations were present in 2/24 (8%) primary PSCC cases. Both cases had an E545A mis-sense mutation in exon 9 (Figure 2). Of the 24 primary PSCC samples, PIK3CA FISH analysis was successful in 23 cases. PIK3CA copy number gain was found in 11/23 (48%) cases. Of the two cases with PIK3CA mutations, one had a coexisting PIK3CA copy number gain. (Figure 3)

### PIK3CA CNS and Hr-HPV analysis

Hr-HPV DNA was detected in 17/24 (71%) of PSCC cases. Hr-HPV 16 was the most prevalent type, seen in 14/17 (82%) of Hr-HPV positive tumours. No significant difference in the frequency of PIK3CA copy number gain between Hr-HPV positive and negative PSCC was seen (p=0.193).

### PIK3CA quantitative RNA expression and correlation with PIK3CA CNS

PIK3CA RNA levels were examined to assess whether PIK3CA copy number gain lead to increased gene expression in affected PSCC samples in comparison to their corresponding normal penile epithelium and samples without genetic alterations at the PIK3CA locus. qRT-PCR was successful in 23/24 PSCC and 14/15 corresponding NPE.

**Figure 4:**
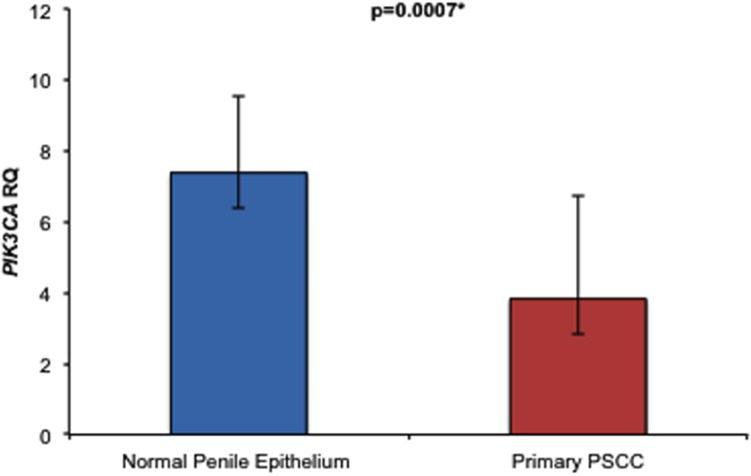
Comparison of overall relative PIK3CA RNA expression levels between NPE and PSCC samples Primary PSCC samples expressed significantly less PIK3CA RNA than NPE samples {3.8 ± 2.9 vs. 7.4 ± 2.2 (p=0.0007)}.

The relative expression of PIK3CA RNA was lower in 8/13 (62%) of PSCC samples in comparison to their matched NPE samples. However, we found the difference in PIK3CA RNA expression between the 13 matched PSCC and NPE samples was not significant (p=0.0803). Overall, the PIK3CA RNA expression of all 23 PSCC samples was lower than in the 14 NPE samples analysed, and this difference was statistically significant (p=0.0007). (Figure 4)

Of the 23 PSCC samples, with PIK3CA RNA quantification, 22 had PIK3CA copy number status available. We found no significant difference in PIK3CA RNA expression levels was found between PSCC samples with or without PIK3CA copy number gain (p=0.4779).

### PI3Kα protein expression and association with PIK3CA CNS and RNA expression

**Figure 5:**
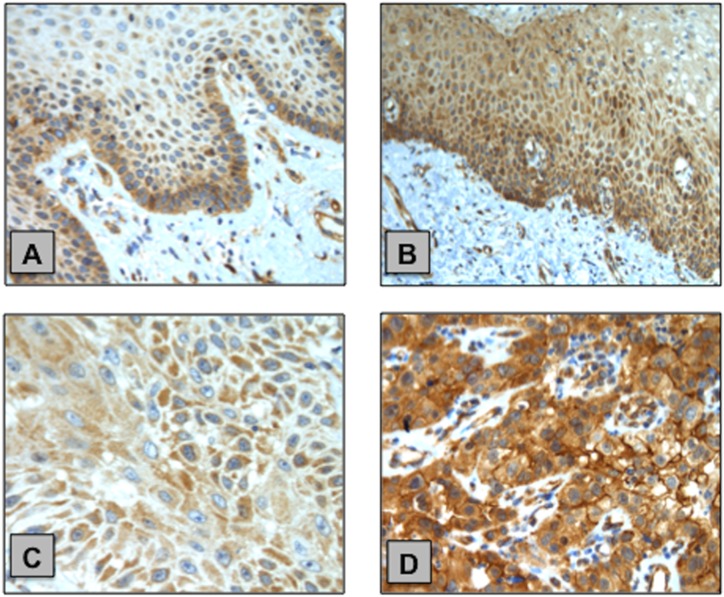
Examples of PI3Kα IHC staining in NPE and PSCC samples **(A)** NPE with weak cytoplasmic staining predominantly localised at the basal layer. **(B)** NPE with moderate cytoplasmic staining. **(C)** PSCC with weak cytoplasmic staining. **(D)** PSCC with strong cytoplasmic staining with areas of membrane localisation of PI3Kα.

PIK3CA expression was further analysed at the protein level (PI3Kα protein) via immunohistochemistry (IHC) and this was possible in 23 PSCC and 15 NPE cases. Overall, we found 18/23 (78%) of the PSCC cohort was positive for PI3Kα immunoexpression compared to 4/15 (27%) of NPE cases and this was statistically significant (p=0.0026). (Figure 5)

However, no significant correlation was found between PIK3CA CNS and PI3Kα immunoexpression (p=0.6404). Nor any significant correlation between PI3Kα immunoexpression and PIK3CA RNA expression in the NPE or PSCC cohorts (p=0.4199 and p=0.2231, respectively)

### Western blot analysis of phosphorylated AKT and mTOR protein expression and association with PIK3CA CNS

In order to evaluate the activity status of the PI3K-AKT-mTOR pathway, analysis of total AKT and total mTOR protein and the three phosphorylated proteins, p-AKT (Thr308), p-AKT (Ser473) and p-mTOR (Ser2448) were investigated using western blot (WB). AKT phosphorylation at Thr308 is facilitated by the PI3Kα protein and results in partial activation of AKT and also gives an indication of the activity status of PI3K protein. Additional phosphorylation at Ser473 leads to full AKT activation and phosphorylation of mTOR at Ser2448 leads to full activation of the mTOR protein. WB analysis was possible in 23/24 fresh frozen primary PSCC samples and 14/15 corresponding normal penile samples.

We found the relative expression of total AKT was lower in 6/14 (43%) PSCC samples in comparison to matched NPE samples, but the majority of matched samples (7/14; 50%) had equivocal total AKT expression. p-AKT (Thr308) protein expression was lower in 12/14 (86%) PSCC samples in comparison to their matched NPE samples; with 4/13 (31%) PSCC samples showing a complete lack of p-AKT (Thr308) expression. Of the matched samples, only 2/14 (14%) PSCC samples had detectable levels of p-AKT (Ser473) whilst 8/14 (57%) NPE samples expressed this protein.

**Figure 6:**
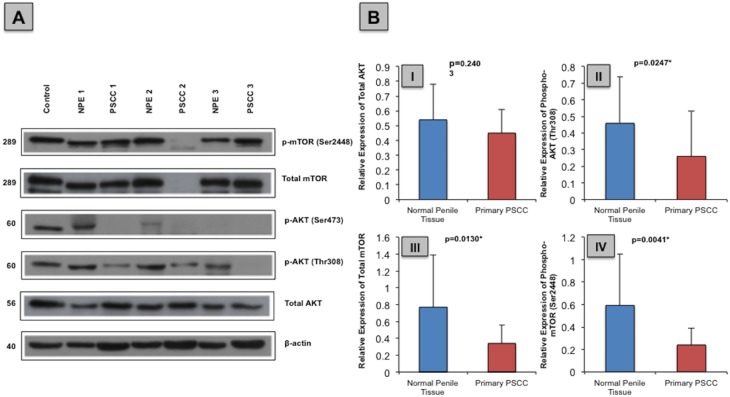
Total and phosphorylated AKT and mTOR protein expression in normal penile tissue in comparison to primary PSCC specimen **(A)** Example immunoblot of total AKT, p-AKT (Thr308), p-AKT (Ser473), total mTOR and p-mTOR (Ser2448) protein expression in NPE and primary PSCC specimens. **(B)** Overall normal penile tissue expressed more (I) total AKT [0.45 ± 0.16 vs. 0.54 ± 0.24; p=0.2403] (II) p-AKT (Thr308) [0.46 ± 0.28 vs. 0.26 ± 0.27; p=0.0247] (III) total mTOR [0.34 ± 0.22 vs. 0.77 ± 0.62; p=0.0130] and (IV) p-mTOR (Ser2448) [0.59 ± 0.46 vs. 0.24 ± 0.15; p=0.0041] than primary PSCC cases.

The majority of PSCC samples had lower expression levels of total AKT in comparison to matched NPE samples (7/12; 58%). In addition, nine out of 12 (75%) PSCC samples expressed lower levels of the p-mTOR (Ser2448) protein compared to their NPE samples. (Figure 6A)

Overall, the relative expression of p-AKT (Thr308), total mTOR and p-mTOR (Ser2448) were significantly lower in PSCC samples than in the NPE samples (p=0.0247, p=0.013 and p=0.0041, respectively). Similarly, PSCC samples expressed less total AKT than NPE samples, though this not statistically significant (p=0.2403). (Figure 6B)

In NPE cases, a significant positive correlation was seen between p-AKT (Thr308) and p-AKT (Ser473) (p=0.0441). No significant correlation was seen between the different phosphorylated proteins and in the expression of p-AKT (Thr308) and p-mTOR (Ser2448) between PSCC samples in respect to PIK3CA CNS.

### Immunohistochemistry of phosphorylated AKT and mTOR, validation of WB analysis and association with PIK3CA CNS

**Figure 7:**
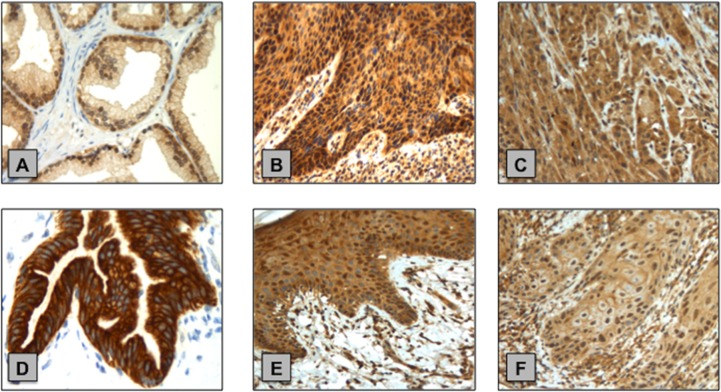
Examples of p-AKT (Ser473) and p-mTOR (Ser2448) IHC staining in normal penile tissue and primary PSCC samples **(A)** p-AKT (Ser473) positive control - prostate cancer tissue. **(B)** NPE with strong cytoplasmic p-AKT (Ser473) staining. **(C)** PSCC with strong cytoplasmic p-AKT (Ser473) staining. **(D)** p-mTOR (Ser2448) positive control - normal pancreas tissue. **(E)** NPE with moderate p-mTOR (Ser2448) cytoplasmic staining and visible nuclear expression. **(F)** PSCC with weak p-mTOR (Ser2448) cytoplasmic and nuclear staining.

Following WB analysis, validation of the p-AKT (Ser473) and p-mTOR (Ser2448) levels in 15/15 NPE and 23/24 PSCC samples were performed using Immunohistochemistry (IHC). (Figure 7)

We found primary PSCC cases (9/23; 39%) had more positive p-AKT (Ser473) cytoplasmic expression than NPE cases (1/15; 7%) however this was not statistically significant (p=0.0562). The converse was true for positive p-AKT (Ser473) nuclear expression with less primary PSCC cases (13/23; 57%) positive than NPE cases (14/15; 93%) and this was statistically significant (p=0.026).

Similarly, PSCC cases had a trend for a lower percentage of cases with positive p-mTOR (Ser2448) cytoplasmic (13/23; 56% vs. 12/15; 80%) and nuclear (7/23; 30% vs. 8/15; 53%) immunoexpression in comparison to NPE cases though this was found to not be statistically significant (p=0.1755 and p=0.1903 respectively). As for WB analysis, we found no significant correlation between the immunoexpression of p-AKT (Ser473) and p-mTOR (Ser2448) between PSCC samples in respect to PIK3CA CNS.

## DISCUSSION

This paper is the first to analyse PIK3CA genetic alterations and their impact on the activation of the PI3K-AKT-mTOR pathway, rather than their individual members, on PSCC and their matched NPE cases. We found fewer PIK3CA mutations (8%), similar to Ferrandiz-Pulido *et al*. [20] who also found a lower prevalence of PIK3CA mutations in 6/65 (9%) PSCC cases. However, we noticed a high frequency of PIK3CA copy number gains (48%) and this suggests that the PI3K-AKT-mTOR pathway may play a role in PSCC carcinogenesis and its primary method of activation could be via PIK3CA copy number gain. This has been shown to be the case in multiple other cancers such cervical cancer [24] and oesophageal cancer [25].

Despite this no significant correlation was found between PIK3CA CNS and PIK3CA RNA expression or PI3Kα immunoexpression. Surprisingly, we found some inconsistencies in the expression levels of PIK3CA between NPE and PSCC samples. For instance, while average PIK3CA RNA expression in fresh frozen samples was higher in NPE than in PSCC the immunoexpression of the PI3Kα protein in the FFPE samples from the same patients was stronger and more common in PSCC samples than NPE. Studies in other cancers are mixed [24, 25], as for example O’Toole *et al*. [26] found in endometrial cancer samples expressed significantly less PIK3C*A* RNA than their matched normal specimen (p<0.01). However, in contrast Redon *et al*. [27] described higher *PIK3CA* RNA expression in both HNSCC with and without *PIK3CA* copy number gain than in adjacent normal tissue.

Two possible explanations for these surprising findings include firstly, tumour cell necrosis, as necrotic areas were evident within in PSCC specimens on histopathological examination. These areas of necrosis may lead to increased degradation of RNA through the release of proteases and RNases into the extracellular space [28, 29]. Secondly, it is possible that NPE has a high PIK3CA RNA expression to aid epithelial proliferation via activation of the PI3K-AKT-mTOR pathway and a lower PI3Kα protein expression due to post-translation regulatory mechanisms, which include proteosomal protein degradation [30], to fine-tune and maintain PI3Kα expression at normal cellular levels [31]. And in PSCC this post-translation regulation of the PI3Kα protein is possibly abrogated leading to high cellular levels resulting in a negative feedback effect and lower levels of PIK3CA RNA expression.

A high number of Hr-HPV positive tumours were identified in our PSCC cohort (17/24; 71%) and this is higher than other recent studies with detection rates varying between 33 to 50% [2, 22]. The higher than expected Hr-HPV rate in this studied is likely due to the small fresh frozen tissue sample size. No correlation was found between Hr-HPV infection and PIK3CA CNS, which suggests this genetic aberration, is not the primary mechanism for the dysregulation of the PI3K-AKT-mTOR pathway in HPV-mediated penile carcinogenesis.

Unexpected results were attained on further downstream analysis of the PI3K-AKT-mTOR pathway to assess its activity status in both PSCC and NPE samples. Firstly, contrary to numerous reports in other cancers, we found no significant association between p-AKT and p-mTOR in either cohort [32–34]. This could be due to the small sample size in this study, which is one of the difficulties with research into rare malignancies such as PSCC, especially on frozen material. Conversely, AKT may be activating alternative pathways instead of mTOR such as activation of IκB kinase, a positive regulator of NF-κB that transcribes anti-apoptotic genes [35, 36] or possibly mTOR is activated by AKT-independent mechanisms in NPE and PSCC such as via the RAS/MEK/ERK pathway, nutrient starvation and hypoxia, among others [37].

Interestingly, the stromal cells in both NPE and PSCC where both positive for phosphorylated AKT and mTOR. This is likely due to their role in propagating the inflammatory process (a key risk factor in PSCC) via activation of the PI3K pathway.

Surprisingly, both WB and IHC protein analysis showed that adjacent NPE expressed more frequently and higher levels of total mTOR (only in WB), phosphorylated AKT and mTOR proteins than PSCC samples. These results suggest that the PI3K-AKT-mTOR pathway is significantly more active in normal penile tissue in comparison to PSCC in our cohort and that this pathway may not be as crucial to penile carcinogenesis as initially thought.

Few studies, have investigated the activation of the PI3K-AKT-mTOR pathway, whilst comparing matched normal and tumour samples and the results are variable. Ferrandiz-Pulido *et al*. [21] found that on immunohistochemical staining, PSCC samples expressed significantly more p-mTOR (Ser2448) than their matched adjacent normal tissue (n=67, p<0.001). In contrast, Fenic *et al*. [38] noted that some of their normal tissue samples expressed similar levels of p-AKT (Ser473) similar to HNSCC cases on WB analysis. Pedrero *et al*. [39] also found that HNSCC and their normal adjacent mucosa had similar expression levels of p-AKT (Ser473) on WB analysis. In addition, Chaux *et al*. [40] demonstrated a generally low level of immunoexpression of p-AKT (Ser473) and p-mTOR (Ser2448) in 112 PSCC cases (up to 10% of cases).

Our findings may be explained by field cancerization due to the chronic inflammatory and HPV risk factors in PSCC [1, 7] and the close resection margins utilised in penile conserving surgery [41, 42]. Therefore, it is possible that the majority, if not all, of our adjacent normal penile tissue have been obtained within a genetically altered field, due to the close resection margins utilised in our institution (<5 mm) and this could account for the increased PIK3CA RNA expression and activity of the PI3K-AKT-mTOR pathway seen. Future studies looking at the epigenetic and genetic differences between non-cancer normal penile tissue, PSCC adjacent normal penile tissue and PSCC would begin to answer the questions of field cancerization in PSCC.

Another possible explanation is induced autophagy within tumour cells to support survival in response to harsh metabolic stress conditions such as a nutrient-limited environment [43]. One of the main regulators of autophagy is mTOR, and it functions in part to supress autophagy in response to nutrient and growth factor availability [44]. So in response to cellular stress, nutrient deprivation and a lack of survival signals, the PI3K-AKT-mTOR pathway is downregulated in PSCC to the result that autophagy is induced and this mechanism is found to be more prominent in cancer cells with defects in apoptosis [45].

No correlation was found between PIK3CA copy number gain, PIK3CA RNA and PI3Kα protein expression or activation of its downstream targets, phosphorylated AKT and mTOR in PSCC. The association between *PIK3CA* amplification/copy number gain and activation of the PI3K-AKT-mTOR pathway varies in the literature. Bertelsen *et al*. [46] found PIK3CA amplification significantly associated with positive p-AKT (Ser473) immunoexpression in 40 cervical cancer cases (p=0.006). Similarly, Agell *et al*. [47] found a strong correlation between high intensity of p-AKT (Ser473) immunoexpression and PIK3CA RNA overexpression and/or genomic copy number gain in 21 prostate cancer cases (p<0.0001). However, similar to our study, Fenic *et al*. [38] found no significant association between p-AKT (Ser473) expression by western blot and PIK3CA copy number gain/amplification or RNA expression in 33 HNSCC cases. This may suggest other PI3K-independent mechanisms play a more prominent role in AKT and mTOR activation in PSCC, such as tyrosine kinases Ack1 and protein tyrosine kinase 6 [48].

Overall, our study shows, despite a high degree of PIK3CA copy number gain in PSCC, the PI3K-AKT-mTOR pathway is significantly less active in PSCC than in NPE tissue, which suggests this pathway is not a key driver in PSCC carcinogenesis. Based on our results the therapeutic targeting of the PI3K-AKT-mTOR pathway in those with advanced PSCC disease is unlikely to produce significant clinical benefit. Future studies will need to focus on the identification of new clinically relevant candidate genes and signalling pathways, which offer prognostic value and the potential for targeted therapeutics in this rare type of cancer.

## MATERIALS AND METHODS

### Ethical approval

Ethics approval for this study was obtained and approved by East London and The City Research Ethics Committee (The Orchid Tissue Bank: 09/H0704/04). Informed consent was obtained.

### Patient samples and clinical data

Twenty-four fresh frozen primary PSCC specimens (20 usual type; 3 basaloid type and 1 mixed usual/basaloid type PSCC) and 15 matched normal adjacent penile epithelium samples and their equivalent formalin-fixed paraffin-embedded (FFPE) tissue blocks (acquisition of the corresponding FFPE block for one fresh frozen PSCC specimen was not possible), were obtained under ethical approval from St George’s Hospital in London with histopathologically confirmed PSCC and with ≥70% tumour purity were included and analysed. All cases were reviewed by a panel of expert uropathologists using the TNM7 staging and classification system for penile cancer (CC, BT and DB).

### Hpv analysis

The presence of HPV DNA was detected by PCR method using SPF10 primers, which amplify a 65-bp fragment of the conserved L1 open reading frame and HPV genotypes identified by the INNO-LiPA HPV Genotyping Extra Assay (Innogenetics NV, Ghent, Belgium) as previously described [3] in 24 PSCC cases.

### Mutational analysis

PIK3CA PCR was performed using a PTC-225 Thermal Cycler, MJ Research (Waltham, MA, US) and specific PIK3CA primers for exon 9 and exon 20 (Sigma Aldrich, UK) were used as >90% of all PIK3CA mutations are clustered in these regions [49]. (PIK3CA primers exon 9 (251 bp): Forward - GAGGGGAAAAATATGACAAAG and Reverse - GAGATCA GCCAAATTCAGTTA; PIK3CA primers exon 20 (190 bp): Forward - TG AGCAAGAGGCTTTGGAGT and Reverse - GGTCTTTGCCTGCTGAGA GT). 300 ng of extracted genomic DNA from the 24 fresh frozen PSCC clinical samples, using a standard phenol-chloroform protocol, were utilised for each PCR run. Product size and PCR reaction specificity was confirmed by 1.5% agarose gel electrophoresis. 1 kb+ ladder (Invitrogen, Life Technologies, UK) was used as a size marker. The amplified PCR products were then sent for automated Sanger sequencing. The presence or absence of mutations within sequenced PCR products of exon 9 and 20 of the *PIK3CA* gene was confirmed using a Nucleotide Basic Local Alignment Search Tool (http://blast.ncbi.nlm.nih.gov/Blast.cgi/).

### Fluorescence *in-situ* hybridisation

PIK3CA copy number status (CNS) was assessed using two BAC clones for FISH probe preparation, which were obtained from the Institute of Cancer Research (Sutton, UK). The first clone (RP11-348P10) was for the control 3p21.3 region and was and the second clone (RP11-245C23) for the gene of interest, PIK3CA located at 3q26.3. The distal 3p21.3 locus was chosen as the control, as this locus showed no copy number change on previous aCGH data [22]. The PIK3CA gene probe was labelled red and the control gene grobe was labelled green.

Tissue microarrays (TMA) were prepared with a manual microarrayer using obtained archival FFPE tissue blocks. Three x 1 mm tissue cores were taken from each tumour. TMA slides were de-waxed in xylene, washed in ethanol and water. Next, they were boiled in pre-treatment buffer (Spotlight tissue pre-treatment kit, Invitrogen) for 15 minutes and digested with pepsin solution (Digest All-3, Invitrogen) for 5 minutes at 28°C. After washing in water and air-drying the slides, the probe was applied following the manufacturer instructions and slides were denatured at 98°C for 10 minutes. The slides were then hybridised at 37°C for 24 hours. A post-hybridization wash in 2 x SCC buffer for 5 min at 42°C was performed, followed by PBS washes. Slides were then dried, counterstained and mounted with Vectashield antifade solution containing DAPI (Vector Laboratories LTD, UK).

All TMA FISH slides were scanned and analysed using the Ariol® SL-50 System (Applied Imaging, San Jose, CA, USA). A minimum of 100 cells with clear hybridization signals was counted per sample. On the basis of FISH results in normal penile epithelium, primary PSCC samples were considered to have copy number gain if the number of FISH signals for PIK3CA was greater than for the normal control probe in at least 20% of counted nuclei. Cores with high background or very weak signals were excluded from the analysis. No signal clusters specific for gene amplification were present.

### Quantitative RT-PCR

Total RNA was extracted from the tumour-rich regions of the fresh frozen PSCC specimens or normal penile epithelium using TRIzol reagent (Invitrogen, Life Technologies, UK) according to manufacturer’s instructions. RNA was quantified using a NanoDrop spectrophotometer (NanoDrop Technologies, Wilmington, US) and cDNA synthesis was performed with 1 μg of total RNA per sample using Moloney Murine Leukemia Virus Reverse Transcriptase, RNase H Minus, Point Mutant kit (Promega, UK).

Quantitative real time PCR (qRT-PCR) were carried out with TaqMan Universal PCR Master Mix (Applied Biosystem, Life Technologies, UK), using TaqMan PIK3CA Expression Assay (Applied Biosystem, Life Technologies, UK; Assay ID - Hs00907957). TaqMan *GAPDH* Expression Assay (Applied Biosystem, Life Technologies, UK; Assay ID - Hs02758991) was used as an endogenous control. PNT2 human cell line (SV40 immortalized normal prostate epithelium) was used as reference to normalise PIK3CA expression in samples from different qRT-PCR runs. All qRT-PCR reactions were prepared according to the manufacturer’s instructions.

The samples in MicroAmp® fast optical 96-well reaction plates (Applied Biosystems, UK) were loaded on an ABI 7500 Real-Time PCR System (Applied Biosystems, UK) and amplified using the following parameters: 40 cycles of 95°C for 10 minutes and 95°C for 15 seconds finally followed by 60°C for 1 minute. Samples were run in triplicates and RNA levels were calculated using the relative quantification method.

### Western blot

WB was performed as previously described [50] using antibodies against total AKT (rabbit monoclonal - AB179463; Abcam, UK) p-AKT (Thr308) (rabbit monoclonal - 2965, Cell Signalling, UK), p-AKT (Ser473) (mouse monoclonal - 4051, Cell Signalling, UK), total mTOR (rabbit monoclonal - AB134903; Abcam, UK) and p-mTOR (Ser2448) (mouse monoclonal - 5536, Cell Signalling, UK). 50 μg of whole cell protein lysates in RIPA buffer were mixed with NuPAGE® LDS Sample Buffer (BioRad, UK) and reducing agent (BioRad, UK) and denatured at 90 °C for 10 min.

Protein samples were separated on either 8% or 10% polyacrylamide gels depending on their molecular weights and transferred onto PVDF membranes (Millipore, UK). Membranes were next blocked with 5% powder milk in Tris Buffer Saline/0.1% Tween-20 and incubated overnight with primary antibodies, followed by secondary peroxidase conjugated antibody (Fisher Scientific, UK) for 1 h at room temperature. Protein detection was done with Immobilon Western Chemiluminescent HRP Substrate (Millipore, UK). β actin levels were used as a loading control (mouse monoclonal - A5441, Sigma-Aldrich, UK).

### Immunohistochemistry

Four-micrometre sections were cut and immunostained using standard heat-induced antigen retrieval methods (pressure cooking for 11 min) with citrate low pH buffer and the ABC kit (Vector Laboratories, PK-6200). Primary rabbit monoclonal antibodies were applied as follow: PI3K p110α (PI3Kα), 1:100 (Cell Signaling, 4249); p-AKT (Ser473), 1:60 (Cell Signalling, 3787); p-mTOR (Ser2448), 1:60 (Cell Signalling, 2976). Positive controls included normal pancreas for PI3Kα, p-mTOR (Ser2448) and prostate cancer for p-AKT (Ser473). For negative control slides, primary antibody step was omitted and only antibody diluent applied instead.

IHC sections were scored semi- quantitatively by a two consultant genitourinary pathologist (GT and DB). The staining pattern of PI3Kα showed mostly cytoplasmic staining with a few sections displaying a degree of membranous specific staining whilst, p-AKT (Ser473) and p-mTOR (Ser2448) showed both cytoplasmic and nuclear staining. The intensity of membranous, cytoplasmic and nuclear staining was measured as: 0 (nil), 1 (weak), 2 (moderate) and 3 (strong). The final membranous and cytoplasmic score was from 0 - 3. For nuclear positivity each core was given an estimated visual score between 0 - 100% representing the percentage of positively stained nuclei. The final nuclear score was deduced by multiplying the percentage of stained nuclei by intensity (0 - 3) to give an expression score from 0 to 300.

The core with the highest score was selected for analysis. Each core was given a score for cytoplasmic and nuclear staining for p-AKT (Ser473) and p-mTOR (Ser2448). Additionally, cores were given a score for m cytoplasmic staining for PI3Kα. For nuclear staining any nuclear expression score >0 was considered to be positive for both p-AKT (Ser473) and p-mTOR (Ser2448) (143). For cytoplasmic staining we classified an intensity score of 0 or 1 as negative and a score of 2 or 3 as positive for PI3Kα, p-AKT (Ser473) and p-mTOR (Ser2448) (170, 171).

### Statistical analysis

Statistical analysis was performed using GraphPad Prism Version 5.03 for Windows. Statistical tests included Fisher’s exact test for PIK3CA CNS comparison with Hr-HPV status and IHC results, Mann-Whitney U tests for PIK3CA CNS correlation with qRT-PCR and western blot data, Wilcoxon signed rank test for qRT-PCR results between PSCC and NPE cohorts and Spearman’s rank correlation test for the correlation between antibodies and qRT-PCR data. All analyses were two sided, p<0.05 were considered significant.

Collection and management of tissue specimen: AA, ES, SK, BT and CC

Performed the experiments: AA and ES

Analysis and data interpretation: AA, ES, GT, Y-JL and DB

Manuscript writing: AA, ES, SK, GT, BT, CC, Y-JL, NW and DB.

## Abbreviations

aCGH: Array comparative genomic hybridization
CNS: Copy number status
FISH: Fluorescence *in-situ* hybridisation
FFPE: Formalin-fixed paraffin-embedded
HPV: Human papilloma virus
Hr: HPV High risk HPV
IHC: Immunohistochemistry
NPE: Normal penile epithelium
p-AKT: Phospho-AKT
p-mTOR: Phospho-mTOR
PIK3CA: Phosphatidylinositol-4,5-bisphosphate 3- kinase
PSCC: Penile squamous cell carcinoma
qRT-PCR: Quantitative real time polymerase chain reaction
TMA: Tissue Microarray
WB: Western Blot.
